# Chronic suppurative otitis media: disrupted host-microbial interactions and immune dysregulation

**DOI:** 10.3389/fimmu.2025.1547206

**Published:** 2025-03-06

**Authors:** Vincent G. Yuan, Anping Xia, Peter L. Santa Maria

**Affiliations:** Department of Otolaryngology-Head and Neck Surgery, University of Pittsburgh Medical Center, Pittsburg, PA, United States

**Keywords:** chronic suppurative otitis media (CSOM), host-microbial interactions, dysbiosis, immune dysregulation, inflammation, polymicrobial infections, innate immunity, adaptive immunity

## Abstract

Recent research has uncovered new mechanisms that disrupt the balance between the host and microbes in the middle ear, potentially leading to dysbiosis and chronic suppurative otitis media (CSOM). Dysbiotic microbial communities, including core pathogens such as persister cells, are recognized for displaying cooperative virulence. These microbial communities not only evade the host’s immune defenses but also promote inflammation that leads to tissue damage. This leads to uncontrolled disorder and pathogen proliferation, potentially causing hearing loss and systemic complications. In this discussion, we examine emerging paradigms in the study of CSOM that could provide insights into other polymicrobial inflammatory diseases. Additionally, we underscore critical knowledge gaps essential for developing a comprehensive understanding of how microbes interact with both the innate and adaptive immune systems to trigger and maintain CSOM.

## Chronic suppurative otitis media: when inflammation disrupts equilibrium

CSOM is a persistent inflammatory condition driven by biofilms that results in perforation of the tympanic membrane, hearing loss, and recurrent or persistent ear discharge (otorrhea) (1–3). A middle ear biofilm is a leading cause of CSOM, eliciting the host’s immune response against the multiple pathogens within the biofilm and ultimately leading to the destruction of the tympanic membrane or eardrum (4). Besides the hearing loss, severe CSOM can negatively impact systemic health by increasing the risk of osteomyelitis, Guillain-Barré syndrome (GBS), and ankylosing spondylitis (5–8). Archaeological evidence suggests that chronic otitis media existed in ancient times and became more common over time due to pathogen infections (9). According to a recent study of ancient skeleton, approximately 2,000 years ago, *Pseudomonas aeruginosa* and other bacteria associated with otitis media were less prevalent than they are today (9).

The studies of the middle ear microbiota have uncovered major changes in microbial community composition that occur as the ear transitions from a healthy state to disease (10–12). Until recent years, the dominant belief was that specific organisms were responsible for the cause of CSOM, with notable examples including *Pseudomonas aeruginosa*, *Staphylococcus aureus*, *Enterobacteriaceae*, and *Klebsiella pneumoniae (* 13). Recent progress from mechanistic investigations collectively suggests that CSOM development is driven by a mix of microbial imbalances and polymicrobial interactions (10, 14–16). Dysbiosis of the middle ear microbiota reflects a shift in the relative abundance of bacterial community components compared to their levels in a healthy state (17, 18). This imbalance leads to changes in host-microbe interactions, which can trigger destructive inflammation (10, 19–21).

The damage to hair cells in the cochlea during later stages of CSOM is well-documented in both human and animal models (22–24). This damage primarily involves inflammatory mediators, endotoxins, and free radicals, as assessed through short-term cultures of isolated outer hair cells (21, 25, 26). However, the initiating mechanisms associated with this damage are not as well understood. Indeed, the mechanisms by which a dysbiotic microbiota triggers uncontrolled or persistent middle ear inflammation, potentially leading to pathological endothelial damage, are not well understood. Furthermore, the sources of dysbiosis and whether it serves as a driving factor or a consequence of the disease remain unclear. Understanding the role and interaction between the host’s immune responses and microbiota in CSOM is difficult. The main aim of this paper is to argue that, while CSOM is clearly an infectious disease, it can also be viewed as a disordered communication between the host and the pathogen.

## *Pseudomonas aeruginosa*: a multifaceted pathogen in CSOM

**Figure 1:**
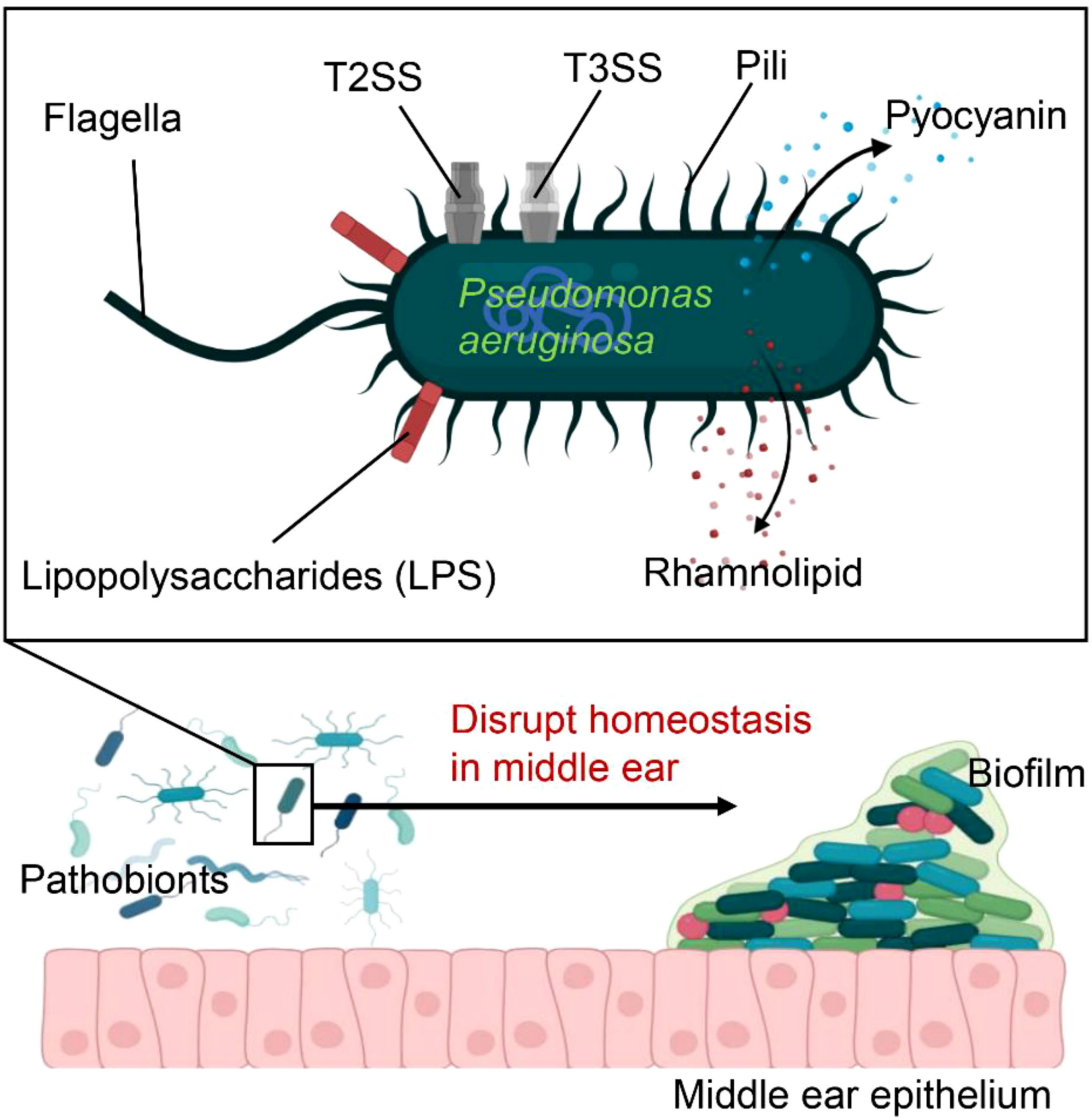
Pseudomonas aeruginosa is equipped with numerous virulence factors that increase its ability to cause disease These include flagella, pili, and lipopolysaccharides (LPS), which facilitate bacterial adhesion and colonization within the host. The secretion systems, T2SS and T3SS, inject effector proteins into host cells, leading to tissue damage. Additionally, toxins such as pyocyanin and rhamnolipid induce immune cell death, thereby promoting bacterial survival. As a key pathogen, Pseudomonas aeruginosa further disrupts homeostasis in the middle ear, promoting bacterial proliferation and biofilm formation.

Pseudomonas aeruginosa has long been linked to CSOM in humans, and our current rodent models further support its role as a key pathogen in the disease (27–29). Nevertheless, *Pseudomonas aeruginosa* not only manipulates the host response but also actively induces inflammation, a trait typically associated with bacteria involved in inflammatory diseases (27, 30). Pseudomonas aeruginosa plays a multifaceted role in immune responses, leveraging various surface structures and secretion systems to compromise host defenses (**Figure 1**). In chronic infections, such as in cystic fibrosis (CF) patients, its flagella and pili, which facilitate motility, are often downregulated to evade immune detection. The bacterium further modulates its immune visibility by altering flagellin expression to reduce activation of Toll-like receptor 5 (TLR5). Its lipopolysaccharides (LPS) also vary, with more acylated forms in chronic infections triggering stronger TLR4 responses. Additionally, *Pseudomonas aeruginosa*’s Type 2 and 3 secretion systems (SS) release virulence factors that damage host tissues and impair immune cells, with toxins like ExoU inhibiting inflammasome activation and inducing necrosis. Other factors, such as pyocyanin and rhamnolipid, induce immune cell death, promoting bacterial survival. Through quorum sensing, *Pseudomonas aeruginosa* regulates these virulence factors to adapt to diverse environments, aiding its persistence in chronic infections (31, 32).

Pseudomonas aeruginosa emerges as a dominant pathobiont in the middle ear due to its advanced survival strategies, including biofilm formation, quorum sensing, and metabolic adaptability (33–35). Unlike many other bacteria, P. aeruginosa produces robust biofilms that shield it from antibiotics and immune clearance, while its sophisticated quorum sensing (QS) system tightly regulates virulence factor expression (36, 37). Additionally, it exhibits metabolic flexibility, thriving in nutrient-poor environments by utilizing diverse carbon sources and efficiently scavenging iron through siderophores like pyoverdine and pyochelin (38–40). These adaptations allow *Pseudomonas aeruginosa* to establish persistent infections, outcompeting other bacterial species in the middle ear.

This persistence of *Pseudomonas aeruginosa* weakens host defenses and alters the bacterial growth environment, causing a harmful imbalance between the immune system and the microbiota (41). Therefore, *Pseudomonas aeruginosa* can worsen disease when the equilibrium is disturbed. Its capacity to exacerbate inflammatory disease through widespread supportive effects within the community has led to its classification as a core pathogen. It is important to recognize that the virulence of a core pathogen such as Pseudomonas aeruginosa persiser cells (non-replicating, metabolically dormant cells with no active transcription or translation) does not depend on pre-existing disruptions in homeostasis (42). This contrasts with pathobionts, which require specific environmental changes in the host, such as a compromised immune system, to induce inflammation (43). Core pathogens have the ability to induce or contribute to the breakdown of homeostasis, meaning that pathobionts typically operate downstream of these core species (10). Bacteria associated with otitis media, including *Pseudomonas aeruginosa*, *Staphylococcus aureus*, *Klebsiella* species, and *Escherichia coli*, are strongly linked to severe inflammatory responses (44, 45). These bacteria not only provoke destructive inflammation but also disrupt the host’s immune response in ways that could potentially enhance the survival of other bystander species (46). *Pseudomonas aeruginosa* is one of the most common pathogens responsible for CSOM, with an incidence exceeding 20% (47, 48). It’s unique ability to drive inflammatory diseases through its widespread impact on community health has led to its classification as a core pathogen, akin to the essential pillars that support bridges (27).

Beyond its intrinsic survival mechanisms, *Pseudomonas aeruginosa* actively suppresses competing bacteria through direct antimicrobial activity and resource competition. It secretes bacteriocins (pyocins) and rhamnolipids, which selectively kill rival microbes (49), while phenazines such as pyocyanin generate oxidative stress that damages other bacteria (50). Moreover, *Pseudomonas aeruginosa* interferes with the quorum sensing of competitors, inhibiting their ability to form biofilms or coordinate virulence (51). By sequestering essential nutrients like iron and modulating the host immune response through proteases and exotoxins (52, 53), it further weakens the local microbiota, ensuring its dominance. These combined factors make *Pseudomonas aeruginosa* a key driver of microbial dysbiosis and prolonged inflammation in the middle ear.

The abundance of pathobionts is not necessarily low; however, they often need changes in the host environment to trigger inflammation. In contrast, a core pathogen does not necessarily rely on disrupted homeostasis to exert its effects (54). Core pathogens have the potential to disrupt homeostasis, meaning that pathobionts generally act downstream of core species. Certain bacteria found in the middle ear, including *S. pneumoniae*, *Haemophilus influenzae*, and *Moraxella catarrhalis*, are closely linked to severe inflammatory responses (55). As such, *Pseudomonas aeruginosa* emerges as a core pathogen. Specifically, it is not only a dominant component of biofilms in the middle ear but also exhibits significant proliferation in diseased CSOM. Deciphering how core bacteria regulate immune responses is crucial for understanding the CSOM microenvironment.

*Pseudomonas aeruginosa* has the ability to modulate adaptive immunity. For example, CD4(+) T cell responses to Pseudomonas aeruginosa differentiate into Th1, Th17, and Th22 subsets (56). If *Pseudomonas aeruginosa* infection is not eliminated in the acute stage, it advances to a persistent infection marked by the formation of a mucoid biofilm. Therefore, it can be hypothesized that *Pseudomonas aeruginosa* exerts core effects by manipulating T cell development in a manner that promotes Th-mediated inflammation.

Since *Pseudomonas aeruginosa* is one of the primary pathogens that cause CSOM, examining the roles of its symbionts could provide valuable insights. Although symbionts have a mutualistic relationship with the host, they share microbe-associated molecular patterns with pathogens. Consequently, they have the potential to trigger inflammation by activating pattern-recognition receptors (PRRs) (57). A recent investigation has found that the occurrence of *A. otitidis* is more frequently linked to chronic otitis media than to acute otitis media. This indicates that *A. otitidis* likely contributes to the continuation of inflammation in otitis media rather than acting as a direct pathogenic agent (58). Research has shown that *A. otitidis* stimulates an immune reaction by promoting the secretion of interleukins from macrophage cell lines in reaction to *A. otitidis (* 59). *In vivo* investigations demonstrated that *A. otitidis*-positive middle ear fluid included similar levels of inflammatory cytokines to those found in *S. pneumoniae*-positive middle ear fluid (60). However, *A. otitidis* often co-occurs with other major pathogens, such as *Pseudomonas aeruginosa* and *Staphylococcus aureus (* 61). The idea that certain commensals can opportunistically trigger destructive inflammation aligns with the growing recognition of previously uncultivable or underappreciated bacteria being linked to otitis media (62). Although most of these species have not yet been cultivated, studies on more accessible organisms have revealed virulence traits indicative of a pathobiont status. For example, the discovery of Alloiococcus within cells suggests potential pathogenic capability (63).

**Figure 2:**
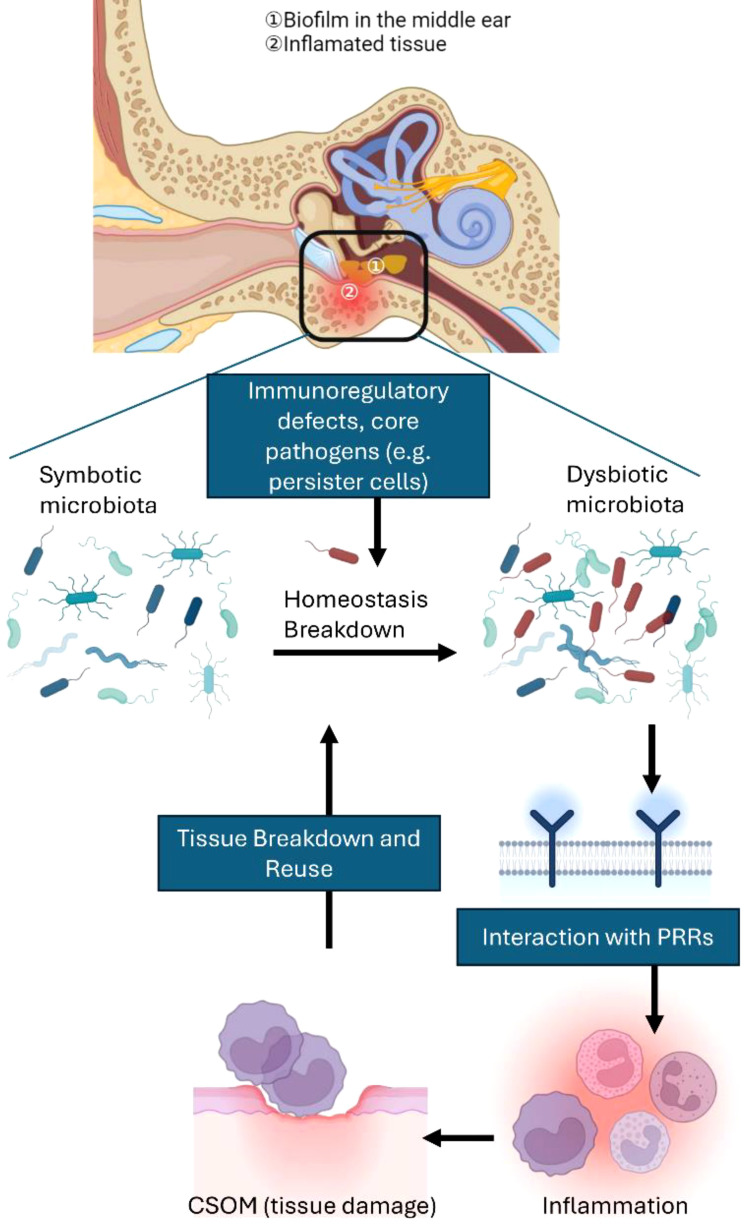
Interactions among multiple microbial species and microbial imbalance in vulnerable hosts contribute to CSOM. A healthy middle ear depends on a regulated inflammatory environment to maintain the balance between host and microbes. However, when there are defects in the host’s immunoinflammatory status or unfavorable environmental factors—defining what can be considered a susceptible host—the balance can shift towards dysbiosis. Dysbiosis occurs when previously benign commensal microbes become proinflammatory pathobionts. Core pathogens (e.g., persister cells) can also play a role in microbial imbalance, even in hosts that do not exhibit clear genetic predispositions. Interactions with pattern recognition receptors (PRRs) frequently drive this imbalance, causing inflammation that ultimately harms middle ear tissue. The resultant tissue damage releases nutrients that further support dysbiosis, creating a vicious cycle of tissue destruction and inflammation. Host susceptibility is a key factor not only in shifting the microbiota from a balanced state to dysbiosis but also in increasing the risk of inflammation that can lead to permanent tissue damage.

As previously mentioned, inflammation provides essential nutrients and greatly impacts the types of otitis media pathogens by supporting bacteria that can utilize the decomposition products (**Figure 2**). On the other hand, pathogens that are unable to adjust to these environmental shifts or are negatively impacted by host inflammation may be outcompeted. The selective proliferation of pathobiont bacteria can trigger a self-perpetuating chain reaction, resulting in additional tissue damage and excessive bacterial proliferation.

## Determinants of susceptibility to CSOM

While the concept of key pathobionts offers valuable insights into CSOM, it also raises several unanswered questions and requires further clarification. For example, the imbalance that promotes the growth of opportunistic microbes may not always result from major pathogens within the microbial community. The balance between microbes and the body can be disturbed by multiple factors, including aging, which is associated with decreased immune regulation and function, elevating the risk of CSOM and hearing loss (64, 65).

Since *Pseudomonas aeruginosa* can be found in healthy people, it’s natural to wonder why it doesn’t always cause CSOM (66). One possible explanation is that certain individuals may possess an innate capacity to withstand or adapt to changes in their middle ear microbial community, preventing it from transitioning from a healthy to an imbalanced state, due to their distinct inflammatory responses (67). Gaining insight into the individual factors that affect vulnerability to microbial immune evasion may provide important knowledge. However, variations in vulnerability might also be attributed to differences in bacterial strains and virulence within the population.

Although ASO has the potential to progress to CSOM, it does not always do so, which suggests that protective host responses might play a role. When ASO does advance to CSOM, it is likely due to the acute infection applying selective pressure that promotes the development of an inflammation-prone microbiota (1). This evolving community may include components, such as persister cells, that can evade or impair the immune system, thus promoting the formation and persistence of a biofilm associated with disease (68).

## Understanding cellular and molecular mechanisms of CSOM

**Figure 3:**
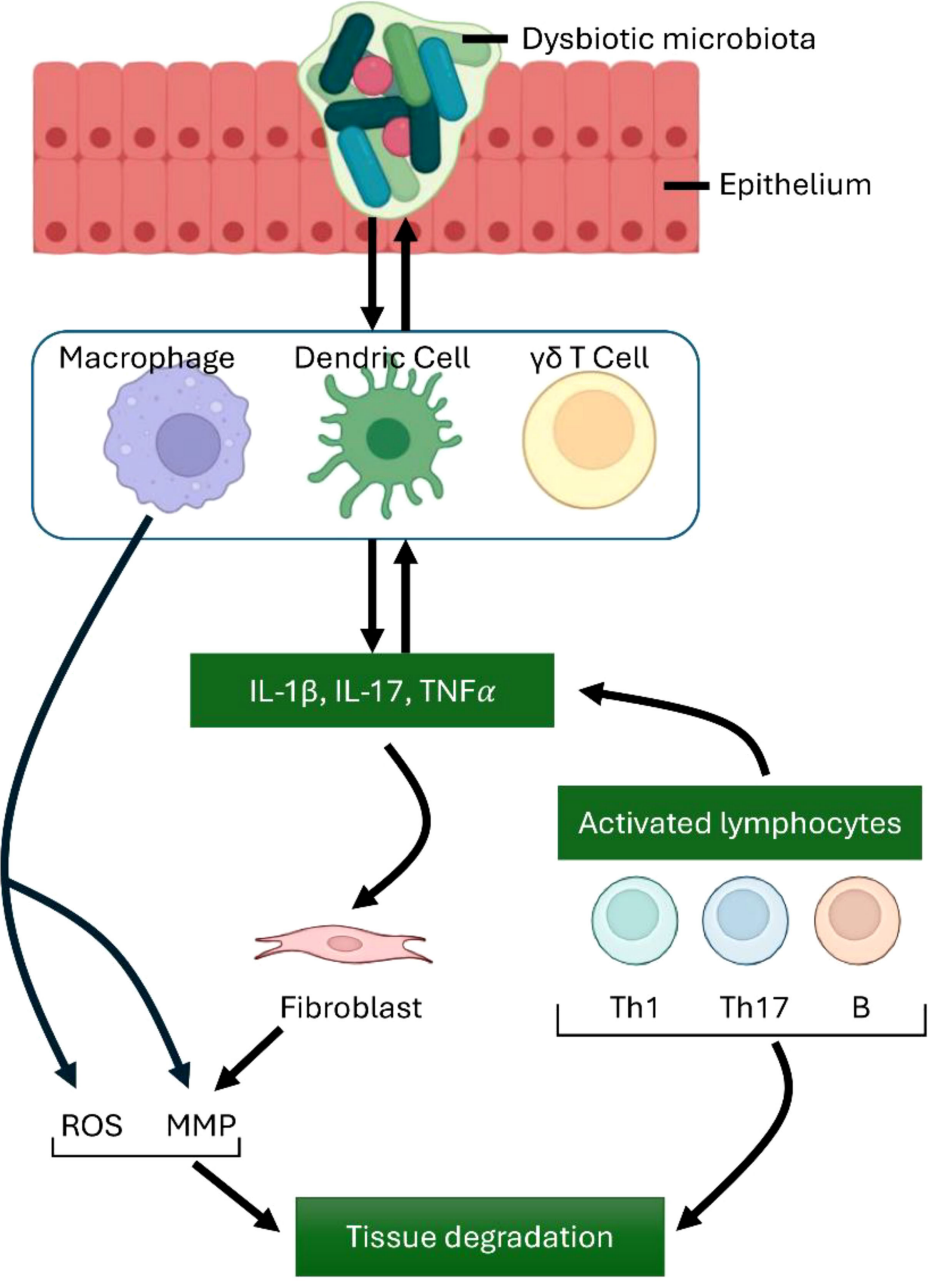
Immune Cell Contributions to Inflammation and Tissue Damage. Macrophages, along with dendritic cells and γδ T cells, produce key proinflammatory mediators, including TNFα, IL-1β, and IL-17.These cells also contribute to the differentiation of Th cell subsets, which in turn amplifies and exacerbates the inflammatory response. IL-17 influences a range of innate immune cells as well as different types of connective tissue cells. Activated macrophages stimulate the production of matrix metalloproteinases (MMPs) and reactive oxygen species (ROS), leading to tissue degradation. Additionally, activated lymphocytes, including B cells and Th1 and Th17 T cells, play significant roles in immune regulation and tissue damage. The interaction between innate and adaptive cells highlights key destructive mechanisms in unresolved inflammation.

One of the primary features of CSOM is the extensive accumulation of macrophages, particularly in middle ear tissues like the junctional epithelium (22). The involvement of macrophages in the development of a chronic condition like CSOM is unsurprising, given their typical association with the extended host reaction to infections (**Figure 3**). Macrophages are increasingly recognized for their involvement in persistent inflammatory disorders, including rheumatoid arthritis (69). Nonetheless, it remains unclear whether the chronic nature of otitis media results from progressive disease deterioration or a cyclical pattern of remission and exacerbation. Additional research is necessary to elucidate the significance of either or both of these frameworks.

**Figure 4:**
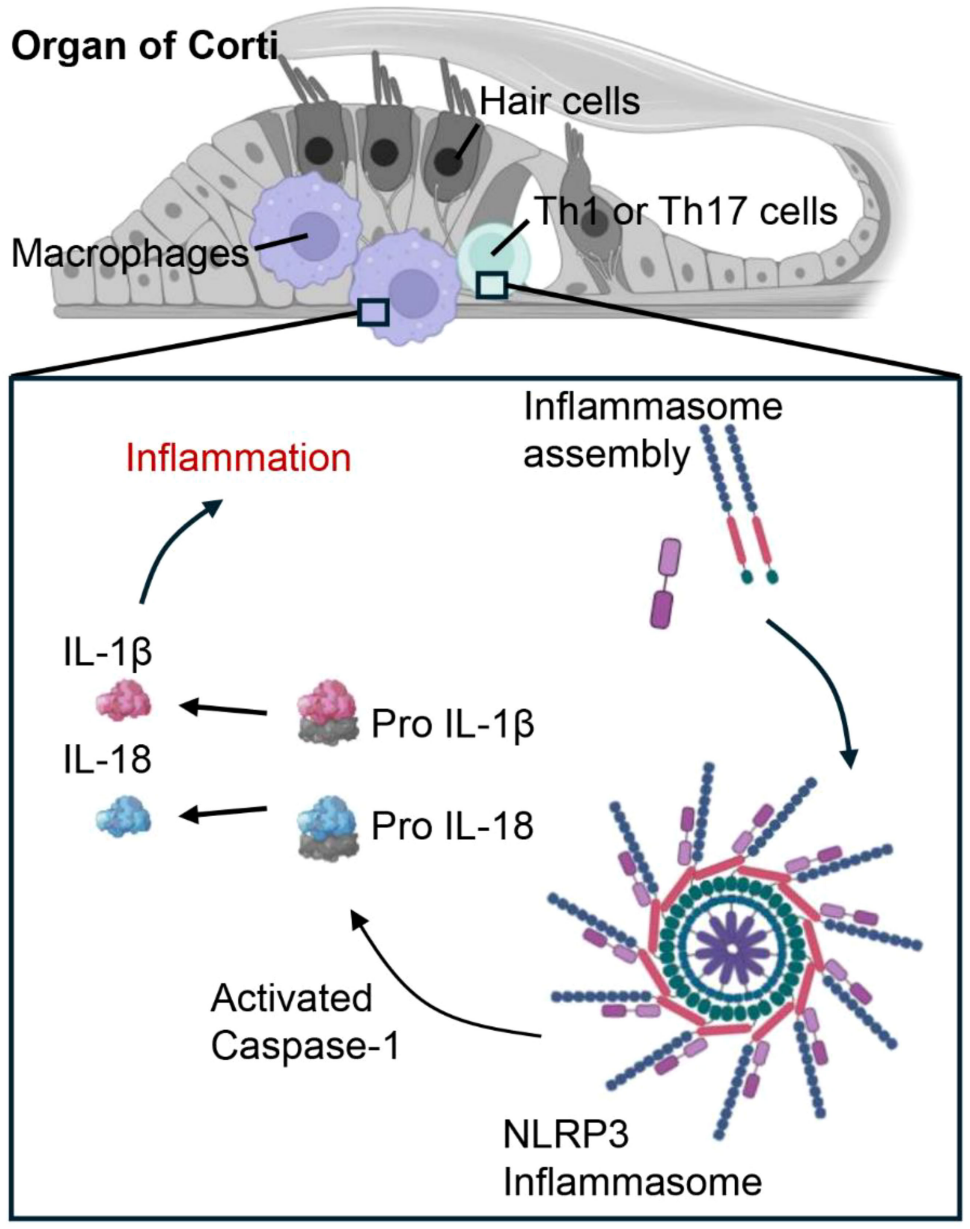
Cochlear macrophages and Th1/Th17 cells promote inflammatory signaling in cases of CSOM via the NLRP3 signaling pathway. In CSOM, the activation of the NLRP3 inflammasome in cochlear macrophages and Th cells initiates Caspase-1, which processes pro-IL-1β and pro-IL-18 into their active forms, thereby intensifying the inflammatory response.

Any alteration in typical macrophage function can disturb the balance of middle ear tissue, potentially resulting in various types of the condition, spanning from early-stage otitis media to CSOM in both children and adults (22). The critical role of macrophages in maintaining middle ear health during inflammation is further reinforced by mechanistic studies in mice. Notably, NOD-, LRR-, and pyrin domain-containing protein 3 (NLRP3) is a key molecule involved in inflammation in the middle ear and hearing loss (70). This multiprotein complex consists of NLRP3, the adaptor protein ASC (apoptosis-associated speck-like protein), and pro-caspase-1. Upon activation by various stimuli in the cochlea (**Figure 4**), including pathogen-associated molecular patterns or danger-associated molecular patterns during CSOM, NLRP3 in macrophages or T helper (Th) cells undergoes conformational changes, leading to the recruitment of ASC and the activation of caspase-1. Caspase-1 subsequently converts pro-inflammatory cytokines, such as pro-IL-1β and pro-IL-18, into their active variants. These cytokines are subsequently released, triggering a robust inflammatory response. In our study, NLRP3-deficient mice exhibited reduced inflammation in chronic otitis media models, suggesting that NLRP3 could be a potential therapeutic target for sensorineural hearing loss (71).

Macrophages can contribute to the destruction of middle ear tissue, which may initiate CSOM, by releasing degradative enzymes such as matrix metalloproteinases and cathepsins, along with cytotoxic substances like reactive oxygen species (72, 73). The cochlea contains a resident population of macrophages that react to injury by proliferating and enhancing the expression of pro-inflammatory molecules. NLRP3 is an essential element of the cochlea macrophages that plays a role in promoting the CSOM. Research has demonstrated that the maturation and activation of macrophages infected with *S. pneumoniae* are contingent upon inflammasome activation, specifically the assembly of NLRP3 inflammasomes (74). Gain-of-function mutations in NLRP3 lead to its abnormal activation and are associated with autosomal dominant systemic autoinflammatory diseases (75). The underlying mechanism indicates that macrophage NLRP3 recognizes a variety of danger signals through a complex pathway involving post-translational modifications and organelle stress. Activated NLRP3 subsequently encourages the oligomerization of ASC into an extensive platform that enables the activation of caspase-1 (70). Furthermore, mice with the NLRP3 D301N mutation exhibit severe inflammation and hearing loss following intraperitoneal lipopolysaccharide injection, indicating that NLRP3 inflammasome activity in macrophages can also contribute to hearing damage through inflammasome-dependent mediators (71). Furthermore, macrophages might contribute to indirect harmful effects by promoting the recruitment of Interferon gamma-producing Th1 cells and Th17 cells, which are implicated in autoimmune responses (76). The recruitment of Th1/Th17 cells mediated by macrophages seems to involve the production of IL-12 subunit p40, p35, and p19 by macrophages, along with the repression of the gene encoding IL-10. As a result, activated macrophages create an environment conducive to a strong Th1 and Th17 response (77).

CD4+ Th1 cells are characterized by their distinctive cytokine production profiles, including IFN-γ and IL-12, and play a crucial role in determining whether an infectious agent is cleared from the body or leads to a chronic infection (78). When Th1 cytokines are necessary for macrophage activation, antigen presentation may be reduced during the early immune response to T-cell-dependent antigens (79). In individuals with otitis media with effusion (OME), Th1 cytokines triggered by IL-12 may affect the initiation or advancement of the condition. Given that IL-12 was found in all patients with OME, it may play a role in the treatment of OME by decreasing late-phase immunological inflammation in the middle ear mucosa caused by lipopolysaccharides, regardless of allergic status or the type of middle ear effusions (MEEs) (80). It is important to highlight that IL-18 or IL-1β production by the NLRP3 inflammasome plays a crucial role in directing the differentiation of CD4+ T cells into Th1 responses (81). Evidence of the inflammasome’s role in promoting autoimmune diseases through Th1 responses has been observed in a mouse model of multiple sclerosis. Consistently, NLRP3-deficient and IL-18-deficient mice showed protection from the disease, accompanied by impaired IFN-γ production (82, 83). Treatment targeting the NLRP3 inflammasome has shown remarkable therapeutic benefits in multiple preclinical models of immune-related diseases, including models of experimental autoimmune encephalomyelitis (84). This inhibitory effect on Th1 differentiation by NLRP3 inflammasome inhibitors, such as MCC950, or anti-IL-1β antibodies was also observed in Nlrp3-/- mouse models (85).

To gain a more integrated understanding of middle ear T cells, examining Th17 cells is crucial. The abundance of IL-17+/Foxp3+ cells is significantly elevated in chronic otitis media, indicating that this may represent an intermediate stage in the transformation process (86). In inflamed middle ears, there is a positive correlation between the number of IL-23+ macrophages and both the severity of inflammation and the frequency of IL-17+ T cells, which constitute the dominant T cell subset (87). Moreover, cytokines that promote Th17 differentiation, such as IL-6, can disrupt the balance between Th17 cells and Tregs (88).

The balance between Th17 cells and regulatory T cells (Tregs) is crucial for maintaining immune homeostasis, particularly in chronic inflammatory conditions such as otitis media with effusion (89). Under normal conditions, Tregs, primarily characterized by the transcription factor Foxp3, suppress excessive inflammation by inhibiting Th17-mediated immune responses through the production of IL-10 and TGF-β (90). However, in CSOM, persistent infection and unresolved inflammation drive an imbalance, skewing the response toward Th17 dominance (89). Inflammatory cytokines such as IL-6 and IL-23 promote Th17 differentiation while simultaneously inhibiting Treg function, further shifting the balance toward a pro-inflammatory state (91, 92). Additionally, chronic inflammation is associated with changes in Treg numbers and function, potentially limiting their ability to counteract Th17-driven pathology (89, 93). The resulting increase in IL-17 production amplifies neutrophil recruitment and tissue damage, creating a self-perpetuating inflammatory loop (94, 95). Targeting the Th17/Treg imbalance with immunomodulatory therapies may provide effective strategies to restore immune equilibrium in CSOM.

Although Th17 cells are known for their strong proinflammatory effects, their overall role in inflammatory diseases triggered by microbes is still unclear. IL-17 can enhance protective innate immunity and activate macrophages. Additionally, IL-22, which is also secreted by Th17 cells, stimulates the production of antimicrobial peptides by epithelial cells (96). Nonetheless, the ongoing presence of Th17 cells in inflamed areas can transform an acute response into a chronic immunopathological condition (97). Interestingly, much like Th1 cells, Th17 cells also exhibit activation of the NLRP3 inflammasome under inflammatory conditions (98). This activation is associated with both disease activity and elevated IL-17A levels. Moreover, inhibiting caspase-1, the IL-1 receptor, or ROS production can reduce NLRP3 inflammasome activation and IL-1β secretion in CD4+ T cells, which subsequently suppresses Th17 differentiation (99). In a related finding, NLRP3 mutant knock-in mice (R258W) develop spontaneous skin lesions. The inflammation seen in these mice is associated with elevated levels of IL-17 family cytokines, including IL-17A, IL-17F, IL-21, RORγt, and IL-22 (100).

Establishing the role of IL-17 in the pathogenesis of human CSOM necessitates the initiation of future clinical trials. Given the high prevalence of CSOM, this topic could also be explored by monitoring patients with CSOM receiving IL-17-targeted therapies for systemic diseases (101). Although IL-17 is primarily recognized as a signature cytokine of Th17 cells, it is also secreted by several other cell types, including innate lymphoid cells (102). The involvement of innate lymphoid cells in CSOM has yet to be investigated. For instance, γδ T cells play a crucial role in IL-17 production, which is strongly induced by TLR signaling activation, likely via the indirect stimulation of phagocytes that produce IL-1β and IL-23 (103).

**Figure 5:**
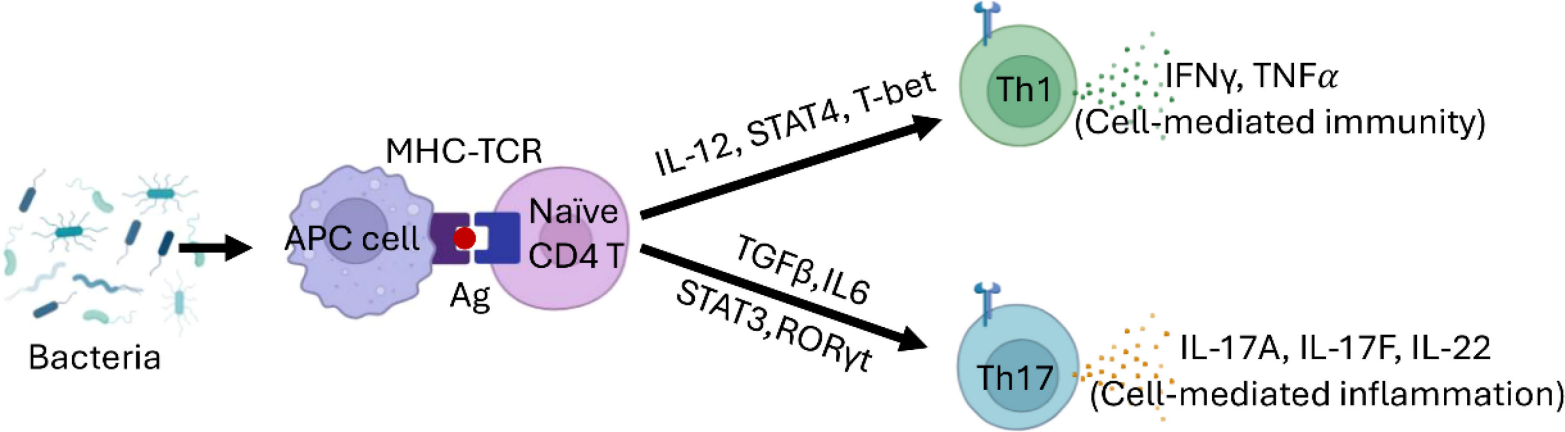
Th Cell Differentiation Model in CSOM. Naive CD4+ T cells differentiate into Th1 cells in the presence of IL-12, T-bet, and STAT4. Th1 cells play a crucial role in cell-mediated immunity, producing IFN-γ and TNFα. In contrast, the differentiation of Th17 cells is driven by TGF-β and IL-6, regulated by STAT3 and RORγt. Th17 cells are vital for host defense against extracellular pathogens and in the context of autoimmune diseases.

Assessing the host response in the middle ear is complex, as antimicrobial activity may also cause inflammatory tissue damage. As a result, defining the exact roles of macrophages and specific effector T cell subsets in otitis media remains challenging. However, macrophages, along with Th1 and Th17 cells, are expected to play a critical role in driving inflammation in the middle ear (**Figure 5**).

## Clinical perspectives

While antibiotics remain a cornerstone of CSOM management, concerns persist regarding recurrent infections, tissue damage, and the rise of antibiotic resistance. These challenges underscore the need for therapies that target inflammation and its signaling pathways rather than solely addressing infection (13). Since middle ear dysbiosis is both a consequence and a driver of inflammation, modulating immune responses may help mitigate tissue damage and restore microbial balance. Experimental therapies targeting early inflammation aim to prevent excessive immune cell recruitment and activation—key contributors to chronic inflammation and hearing loss (104, 105). In animal models, IL-1 receptor antagonists and NLRP3 inhibitors like MCC950 have shown promise in reducing inflammation and slowing disease progression, paving the way for clinical trials in human CSOM patients (71, 106).

Beyond immunomodulation, advances in personalized medicine may refine treatment strategies by identifying patient subgroups most likely to benefit from specific therapies based on biomarkers of immune dysregulation (107). Additionally, novel drug delivery systems, such as nanoparticle-based formulations that selectively target immune cells, offer a promising strategy to enhance treatment precision while minimizing systemic side effects (108). Improving diagnostic tools, including molecular profiling of inflammatory mediators and microbiome analysis, may facilitate early detection and tailored interventions (109).

Future clinical research should integrate these novel therapeutic avenues with improved diagnostic and management strategies to develop more effective, individualized treatments for CSOM. A multidisciplinary approach combining immunology, microbiology, and bioengineering holds the potential to shift CSOM management from symptomatic relief to precision-targeted interventions that address the underlying inflammatory and immune dysregulation driving disease progression.

## Concluding remarks

The maintenance of middle ear homeostasis serves as a protective barrier that separates the host from the microbiota, effectively managing occasional microbial invasions through prompt immune system responses. This controlled state of inflammation reflects a protective response by the host. The onset of chronic CSOM arises from the combination of a disrupted microbiota and a vulnerable host, resulting in intricate inflammatory responses. Dysbiotic microbial communities often participate in cooperative interactions that improve their capacity to colonize, secure nutrients, and persist within an inflamed setting, increasing their adaptive advantage. Although key pathogens like *Pseudomonas aeruginosa* can interfere with the host’s defenses and lead to homeostatic imbalance, certain other bacteria may function as pathobionts, triggering damaging inflammation that engages both the innate and adaptive immune systems. From a microbial perspective, inflammation is vital as it supplies essential nutrients, though it can also lead to collateral damage to middle ear tissues. Therefore, targeting inflammation is central to treating CSOM; however, a comprehensive model of pathogenesis still needs further research.
